# Determinants of Habitat Selection by Hatchling Australian Freshwater Crocodiles

**DOI:** 10.1371/journal.pone.0028533

**Published:** 2011-12-07

**Authors:** Ruchira Somaweera, Jonathan K. Webb, Richard Shine

**Affiliations:** School of Biological Sciences, University of Sydney, Sydney, New South Wales, Australia; Macquarie University, Australia

## Abstract

Animals almost always use habitats non-randomly, but the costs and benefits of using specific habitat types remain unknown for many types of organisms. In a large lake in northwestern Australia (Lake Argyle), most hatchling (<12-month-old) freshwater crocodiles (*Crocodylus johnstoni*) are found in floating vegetation mats or grassy banks rather than the more widely available open banks. Mean body sizes of young crocodiles did not differ among the three habitat types. We tested four potential explanations for non-random habitat selection: proximity to nesting sites, thermal conditions, food availability, and exposure to predation. The three alternative habitat types did not differ in proximity to nesting sites, or in thermal conditions. Habitats with higher food availability harboured more hatchlings, and feeding rates (obtained by stomach-flushing of recently-captured crocodiles) were highest in such areas. Predation risk may also differ among habitats: we were twice as likely to capture a crocodile after seeing it in open-bank sites than in the other two habitat types. Thus, habitat selection of hatchling crocodiles in this system may be driven both by prey availability and by predation risk.

## Introduction

Natural habitats are highly heterogenous in space and time, and living organisms distribute themselves in non-random ways across that mosaic [1], [2], [3]. Habitat-selection behaviours by animals result from species-specific proximate responses to a wide range of abiotic and biotic cues that predict habitat suitability [4], [5], [6]. Such responses presumably have evolved through selective forces imposed by the costs and benefits of occupancy of alternative habitat types, and understanding the nature of such costs (e.g. predation risk) and benefits (e.g. food availability) can clarify the underlying causes for biotic distributions [7], [8]. However, although the importance of costs and benefits has been shown both theoretically and in the laboratory, relatively few field studies on vertebrates have quantified habitat-selection trade-offs between food and safety (see [9] for a review).

The costs and benefits of alternative habitat types depend upon the attributes of the organism in question, and patterns of habitat utilisation frequently differ even between closely related organisms – for example, habitat use may differ between the sexes within a population [10] or change seasonally or ontogenetically within the lifetime of a single individual [11], [12], [13]. Habitat requirements of the most vulnerable life-stages within a population are of particular interest for management purposes. For example, if neonates or reproducing females require specific habitat types, then maintaining such areas is essential for effective population management [14], [15], [16]. For most kinds of animals, we do not understand habitat requirements in detail.

As in many other vertebrates, the earliest life-history stages (eggs and hatchlings) of crocodilians experience much higher mortality rates, from a range of causes, than do larger, older conspecifics [17]. Hatchling crocodiles often use habitats non-randomly; for example, young Australian freshwater crocodiles (*Crocodylus johnstoni*) typically select shallow water [18], [19], [20] but the consequences of this non-random habitat use for predation risk and/or feeding opportunities are unclear [20]. We examined the habitat use of hatchling freshwater crocodiles in a large impoundment (Lake Argyle) in tropical northwestern Australia, comparing the three main habitat types available in terms of their usage by crocodiles, and the potential costs and benefits of that usage. Specifically, we tested hypotheses that hatchling crocodiles may disproportionately be found in some habitats rather than others because of proximity to nesting sites (if dispersal is risky or difficult) [21], [22]; thermal regimes (suitable sites may provide metabolic benefits) [23], [24], [25]; food supply (prey-rich sites may enhance feeding rates); and/or vulnerability to predation (crocodiles may avoid habitats where they are less likely to be able to evade a predator).

## Materials and Methods

This project was conducted under the approval of the Animal Ethics committees of the University of Sydney (approval No. L04/9-2009/3/5108) and the Department of Environment and Conservation in Western Australia (approval No. DEC AEC 32/2009/research permit No. SF007535).

### Study species

The freshwater crocodile *Crocodylus johnstoni* is endemic to tropical mainland Australia. Juvenile freshwater crocodiles mostly consume invertebrates, with fish and other vertebrates becoming more important prey for larger size-classes [18], [19], [20]. In Lake Argyle, female *C. johnstoni* oviposit in August-September, with the eggs hatching in late October though December. Hatchlings are seen in abundance from this time, initially in groups (often accompanied by an adult), but these crèches are no longer evident by March-April. In this paper, we define ‘hatchlings’ as crocodiles in their first year of life (i.e., <12 months old). We surveyed hatchling distributions during March-April, to ensure that we were studying the results of habitat selection by hatchlings rather than by their guardians. Hatchlings emerging from natural nests at Lake Argyle ranged from 9.8 to 13.1 cm (mean  =  11.7 cm) snout-vent length (SVL) (Somaweera unpub. data). For the current study, we define “hatchlings” as crocodiles <25cm SVL. Based on growth rates of Lake Argyle crocodiles, such animals are less than 12 months old (Somaweera unpubl. data).

### Study area and habitat types

**Figure 1:**
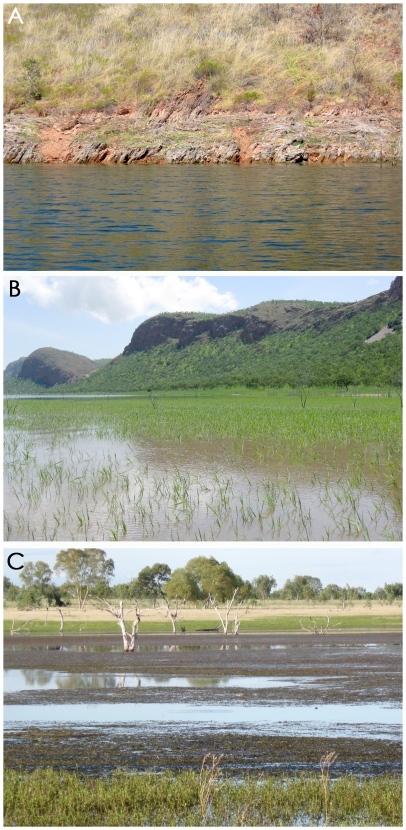
The three main habitat types used by hatchling *Crocodylus johnstoni* at northern Lake Argyle. A- open bank, B- grassy bank, C- floating vegetation mats. Open banks lack riparian vegetation, grassy banks have a shoreline with emergent grasses while floating mats of vegetation comprise submerged and floating aquatic vegetation along the shoreline.

Lake Argyle, in seasonally arid northeastern Western Australia (16° 29′S, 128° 75′E), is the largest man-made lake in Australia (880 km^2^ at normal water level). The lake contains >30,000 non-hatchling *C. johnstoni* but few saltwater crocodiles (*C. porosus*) [26], [27], [28]. In the course of a broad-ranging ecological study on this freshwater crocodile population, we rarely saw hatchling crocodiles in deep water. Instead, we saw them in the following three discrete habitat types (see Figure 1 for photographs of examples).

1. open banks - sandy or rocky shorelines without riparian vegetation; hatchlings usually seen in shallow water close to shoreline.
2. grassy banks - shoreline with emergent grasses including *Eriachne sulcata, Echinochloa kimberleyensis* and/or *Oryza australiensis* extending from the banks into >1m deep water, and
3. floating mats of vegetation - little vegetation on banks, but thick mats of submerged and floating aquatic vegetation comprising *Najas graminea, Hydrilla verticillata, Potamogeton tricarinatus, Valisneria spiralis, Myriophyllum verrucosum, Chara* sp. and/or *Nymghoides indica* growing in relatively deep (>1m) water.

To measure availability of habitats, each habitat type was delineated on a 1∶20 000 topographical map during ground surveys and measured to determine the amount of habitat type available on each study site. The sites we used were located far from any areas with tourist activity, so the level of human disturbance among sites is similar.

### Analysis of habitat use by hatchling crocodiles

We conducted spotlighting surveys from 27 March to 8 April 2010 (when hatchlings were 4-5 months old), from a boat at night (1800–2300 h). We surveyed 30km of shoreline (6km each in five sites), and counted the number of hatchling crocodiles seen, and the number captured, in areas representing the three habitat types. Our analyses are based only on animals that we approached closely enough to estimate their body sizes.

### Thermal attributes of each habitat type

Hatchling crocodiles were mostly seen in water, so we focus attention on thermal characteristics at 10–15 cm and 50 cm below water at 1m and 5m from the shoreline in each habitat type. We deployed three thermochron iButton temperature loggers (programmed to record temperatures at 60 min intervals) at each water depth and each distance from the shore in each habitat type for 14 days.

### Measurement of prey availability through sweep netting

We used sweep-net surveys to assess the abundance of potential prey in 26 replicate areas within each of the three habitats. Sweep-netting was done two days prior to collection of hatchlings for stomach samples. At each of these sites, a 590×680×560 mm net (mesh-size 1×2 mm) was scooped 10 times on land (from the water's edge to 2m from the water, all at ground level) and another 10 times over an equivalent area in the water (i.e., to cover the areas where hatchling crocodiles feed: R. Somaweera per. obs.; [18]). Thus, we collected 26 samples (520 sweeps) from each habitat type. Sweep-netting was conducted at 1700–1830 h, without any artificial light. We identified the animals to order level and counted the number of individuals in each size class (in ‘target sizes’ according to the template mentioned below). We restricted these analyses to taxa that were actually consumed by crocodiles (>5% of prey in the stomach contents samples: see below).

### Analysis of crocodile food intake via stomach-flushing

During surveys in March-April 2010, we hand-collected 99 hatchlings (<25 cm SVL), with equal numbers (n = 33) from each of the three habitat types. We stomach-flushed the animals soon after capture. A rubber ring (secured with a rubber band) held the mouth open while a water-lubricated silicone tube (4mm inner diameter) was inserted to the stomach. Water was then gradually pumped in using a 60-ml syringe. The tube was moved back and forth to stir the contents and then was gradually extracted while the crocodile was inverted and its abdomen massaged. The procedure was repeated until the water was devoid of food particles (2–4 times); regurgitated stomach-content samples were seined through cheesecloth, preserved in 70% ethanol, and stored for later identification. We marked each crocodile by tail-scute clipping and used a flexible tape to measure total length (TL), snout vent length (SVL), and head length (HL) to the nearest 0.1 cm. Mass was recorded to the nearest gram using an electronic balance.

We separated the prey samples into freshly-ingested (<24 h prior to capture) and old (ingested >24 h before capture) items, following criteria from [29]–[31]. Only freshly ingested material was considered in our analysis, to improve certainty of identification, and reduce the possibility that prey were captured in a habitat different from that in which we found the crocodile. We analysed the fresh prey samples to identify the taxa and mass (to 0.1 g) of each prey type in each stomach sample (after washing and air-drying for 30 min to remove excess water). The size of the prey items was scored with a two-dimensional template of ‘target size’ (the smallest rectangle in the template that can accommodate the maximum presentable area of the prey, excluding appendages such as antennae [18]. When appropriate, data with non-normal distributions were log (*x* + 1) transformed to achieve variance homogeneity before statistical analysis.

### Vulnerability to predation

We attempted to capture every crocodile that we sighted. The same spotter, catcher and the driver in the same boat were used for all surveys. Significant spatial differences in our capture success would mean that crocodiles were harder to catch in some places than others, hinting that natural predators might encounter similar difficulties.

## Results

### Habitat use by hatchling crocodiles

**Figure 2:**
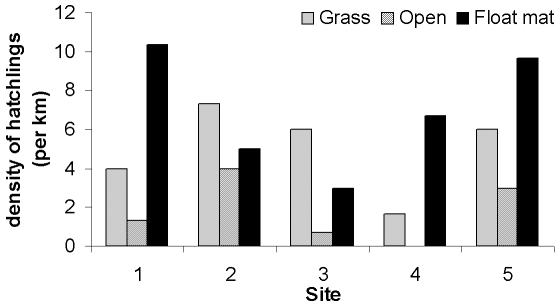
The density of yearling (4 to 5 month old) *Crocodylus johnstoni* in five study sites in northern Lake Argyle. Higher densities of hatchlings were found in floating vegetation mats and grass banks compared to open banks in each study site.

Grass banks and floating vegetation mats comprised 25% and 24% of the lakeshore in our five study sites, respectively, and the rest were open banks. Crocodile numbers were similar among the five study sites (ANOVA, F_4,10_ = 0.61, p = 0.66), but crocodiles were not distributed evenly among the three habitat types. Despite their higher availability, open banks harboured fewer crocodiles than did either floating mats or grassy banks (F_2,12_ = 5.85, p = 0.02; posthoc Fisher's PLSD test, p<0.05 for open banks vs both of the other categories). Although comprising <25% of the available area, floating vegetation mats were the habitat most often selected by hatchling *C. johnstoni* at Lake Argyle (Figure 2).

In the sample of crocodiles caught for stomach-flushing, animals from the three habitat types had similar mean head lengths (mean 61.9±13.5mm; F_2,96_ = 0.04, p = 0.96), snout-vent lengths (mean 181.6±35.3mm; F_2,96_ = 0.2, p = 0.82), total lengths (mean 384.7±78.2mm; F_2,96_ = 0.01, p = 0.99) and body masses (mean 175.2±107.9g; F_2,96_ = 0.32, p = 0.73).

### Proximity to nesting sites

**Figure 3:**
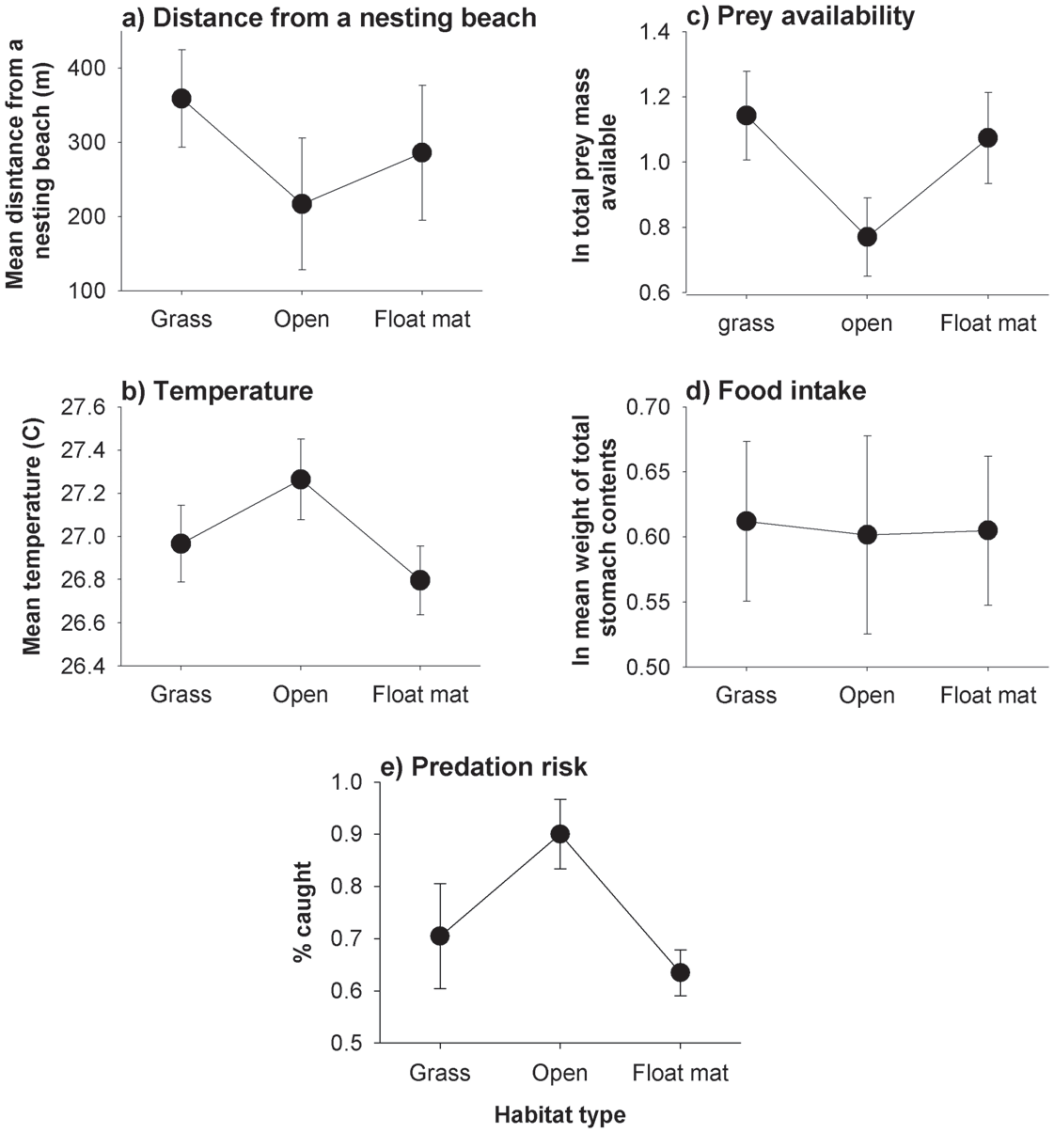
Habitat and prey selection by hatchling *Crocodylus johnstoni* found in three habitat types (grassy banks, open banks and floating grass mats) at Lake Argyle. The three different habitats are found at similar distances from nesting beaches (a) and are similar in thermal characteristics (b). Despite lesser food availability in some habitats (c) the total food intake of hatchlings was similar among habitats (d). However, hatchlings that we found in open banks were easier to capture, suggesting higher vulnerability to predation (e). All graphs show standard errors.

Each habitat type occurred within each of our five sites, with no significant difference among the three habitat types in their mean distance from nesting beaches (F_2,12_ = 0.74, p = 0.49, Figure 3a).

### Thermal attributes of each habitat type

Mean water temperature did not significantly differ among the three habitats (F_2,33_ = 1.83, p = 0.18, Figure 3b), nor at different distances from shoreline (habitat*distance: F_5,30_ = 1.74, p = 0.15) nor at different depths (habitat*depth: F_5,30_ = 1.3, p = 0.29).

### Prey availability in each habitat type

Overall, hemipterans were the most abundant potential prey type, followed by odonates, coleopterans and arachnids (Somaweera unpubl. data). Sweep-netting revealed that grass banks had the largest number of potential prey items; open banks had fewer prey than did either grassy banks or floating mats (F_2,75_ = 28.33, p<0.0001; Fisher's PLSD test, p<0.05 for open banks vs others) and also a lower diversity of morpho-species per sample (F_2,75_ = 16.86, p<0.0001; Fisher's PLSD test, p<0.05 for open banks vs others). Although the modal size of prey items was larger in open banks than the other habitats (F_2,75_ = 12.76, p<0.0001; Fisher's PLSD test, p<0.05), the total mass of potentially available prey was highest in grassy banks and lowest in open banks (F_2,1596_ = 14.83, p<0.0001; Figure 3c).

### Food intake

Of the 99 animals examined, six had empty stomachs (four from open banks and one each from floating mats and grass banks). Stomach contents yielded 592 identifiable prey items that included spiders, aquatic insects, terrestrial insects, crustaceans, fishes and anurans (Figure S1). Crocodiles from open bank habitats had a lower average number of prey items (F_2, 96_ = 4.17, p = 0.02), fewer prey taxa (F_2, 96_ = 6.35, p = 0.003) and fewer types of food items (morpho-species) per stomach (F_2, 96_ = 8.11, p<0.001) than did animals from grassy banks or vegetation mats (Fisher's PLSD test p<0.05 for open banks vs others for both comparisons). However, the modal sizes of prey items consumed by hatchlings in open banks were larger than those in the other habitats (F_2, 90_ = 4.07, p = 0.02). Overall, the mean mass of stomach contents did not differ significantly among crocodiles from the three habitat types (F_2,96_ = 2.26, p = 0.11; Figure 3d).

The total number of food items per stomach was positively correlated with the density of hatchlings in a survey site (F_1,86_ = 8.42, p = 0.004). This pattern was similar among habitats (interaction habitat* crocodile density F_2,86_ = 1.94, p = 0.15). The total mass of food items per stomach also was significantly correlated with the density of crocodiles (interaction habitat* density F_2,86_ = 1.29, p = 0.28, so deleted; crocodile density vs stomach contents mass, F_1,97_ = 4.67, p = 0.03). That is, hatchling crocodiles were concentrated in areas that provided high rates of food intake.

### Vulnerability to predation

We saw 412 hatchlings, and captured 270 of them. The number of animals seen vs caught differed among the three habitat types (Logistic regression, χ^2^ = 8.19, df = 2, p = 0.017; Figure 3e): we failed to catch 38% of hatchlings seen in floating mats and 35% of those seen in grassy banks, but missed only 19% that were seen in open–bank habitat.

## Discussion

The habitats that juvenile vertebrates select early in life can profoundly affect their access to food, their growth rates, their risk of predation, and their future survival [32], [33], [34], [35]. Our results show that in Lake Argyle in tropical Australia, hatchling freshwater crocodiles used habitats non-randomly. Most hatchlings used floating vegetation mats and grassy banks, and seldom used open-bank habitats. A similar preference for areas with aquatic vegetation is common in hatchling crocodilians of other species also [36], [37].

In crocodilian species with prolonged parental care, the number of young animals in a habitat may be determined by maternal habitat selection [38]. However, the bias in habitat use in our site was not due to habitat selection by parents. During our sampling period, the hatchlings had dispersed from their crèches and were not guarded by adults. As in some other crocodilian species (e.g. *C. acutus* [32], [39]) and populations (e.g. *A. mississippiensis* [40]), pods of *C. johnstoni* disband within four weeks of hatching (Somaweera pers. obs).

Post-hatching crèches in crocodilians commonly occur close to the nesting site (e.g. [32], [41] but see [42], [43] for counter examples) and hatchlings sometimes remain near their nest of origin for more than a year [44], [45]. Because nesting beaches were randomly located among the different habitat types within our sites, proximity to nesting areas cannot explain the spatial distribution of young crocodiles in our study. Neither can disturbance by humans, because we were the only human visitors to most of these sites. Thermal characteristics did not differ significantly among habitat types, suggesting that this factor cannot explain non-random habitat use during the study period either. Water temperatures at Lake Argyle change seasonally with air temperatures, and are lower during the dry season (April to October) and warmer during the wet season [46]. Nonetheless, our data suggest that inter-habitat variations in temperatures are likely to be minor.

Two of the factors that we measured differed among habitat types: prey consumption rates and exposure to predators. Spatial heterogeneity both in food supply and in the risk of predation can be important determinants of habitat use ([9], but see [47], [48]). For example, juvenile *Caiman crocodilus* may select habitats based on availability both of insects and of sheltering vegetation [38]. Increased effort to acquire resources reduces the probability of death by starvation but increases the probability of death by predation [47].

In our study both the total number of food items and the total mass of fresh food ingested by hatchlings were positively correlated with hatchling density, suggesting that hatchlings select habitats that maximise their feeding rates. Although previous studies have assumed that crocodilian hatchlings select habitats that are rich in food [38], [49], [50], our field data may be the first to actually demonstrate such a link. If the growth rates of hatchlings are related to food availability [50], hatchlings may concentrate in food-rich areas [32]. However our results show that despite differences among habitats in the types and amounts of food available, hatchlings were able to maintain similar overall nutritional intakes. The lower number and diversity of prey items consumed in open-bank habitats was balanced by the higher mean mass per prey item; and presumably, the lower densities of hatchling crocodiles reduced intraspecific competition in such sites. Thus, overall nutritional input (presumably the most critical parameter for crocodile fitness) did not differ among the three habitat types, supporting the hypothesis that animals assort themselves among alternative habitat types at densities such that average food consumption rate is similar in all habitats [1]. Future work could usefully examine potential differences in food quality among habitats.

Food availability may not be the sole determinant of hatchling distributions. For example, hatchlings were less common in grassy banks than in vegetation mats, despite grassy banks harbouring more potential prey items. Subaquatic habitats with more emergent vegetation (equivalent to grassy banks in our study) may support more insect biomass, and thus provide more food for hatchling crocodilians [50]. Sheltered habitats also may minimize hatchlings' exposure to thermal extremes and wave action [39]. More importantly, hatchlings may be more vulnerable to predation in open habitats, based on the way that our own capture rates differed among habitat types. Although hatchlings inhabiting open banks could dive more easily than in the other habitats (unimpeded by vegetation), we nonetheless found them easier to capture because they could not escape to cover (hatchling crocodilians may seek shelter when under threat [51]). Similarly, hatchlings from grassy banks were easier to catch than those in vegetation mats. If vulnerability to human approach can be used to assess “natural” predation risk [52], hatchling crocodiles in more open habitats may face a higher risk of predation. However, our capture attempts provide a useful proxy only for certain types of predators, and may not realistically simulate some of the predatory taxa to which hatchling crocodiles are potentially vulnerable – especially, those that approach from underwater (e.g. larger crocodiles, fish, turtles), the air (e.g. raptors, waders) or the land (e.g. dingoes, goannas), by day as well as by night ([53]; [54]; Somaweera in prep.). Although it is difficult to quantify predation risk by a large guild of predators on nocturnal, aquatic species [55], quantitative information on the importance of alternative predators in this system could help to further refine habitat-specific estimates of predation risk.

Animal populations are limited by both food and predators, because food availability affects the probability of death by predation and predator density affects the probability of death by starvation [46]. Most environments represent a mosaic of different habitats that can provide different levels of these resources [56]. In some situations animals are distributed across habitats proportional to food availability (e.g., guppies *Poecilia reticulata* [57]; armored catfish *Ancistrus spinosus* [58]; current study), but if predation risk varies among habitats, prey will not necessarily select habitats based solely on the energetic return [9].

In conclusion, our results suggest that the selection of vegetated habitats by young freshwater crocodiles may be a function of both benefits (food availability) and costs (predatory risk). Given that human-regulated water levels in Lake Argyle are stable during most of the year, the costs and benefits of occupying alternative habitats may be more stable seasonally than is the case in many riverine habitats occupied by this species. Proximity to nest-sites, suitable thermal regimes or avoidance of human disturbance cannot explain the non-random patterns of habitat use that we documented. The higher concentration of hatchling crocodiles in refuge habitats such as vegetation mats and grassy banks emphasizes the importance of these habitats for this critical life stage. Variation in the availability of these habitats through space and time thus may influence crocodile recruitment; and accordingly, management of this system needs to ensure that such habitats are retained in order to provide the resources important to the youngest life-history stages.

## Supporting Information

Figure S1 **Stomach contents of 4-5 month freshwater crocodiles (** ***Crocodylus johnstoni*** **) from Lake Argyle, Western Australia.**
(XLS)
